# High Sensitivity Surface Plasmon Resonance Sensor Based on Periodic Multilayer Thin Films

**DOI:** 10.3390/nano11123399

**Published:** 2021-12-15

**Authors:** Haoyuan Cai, Shihan Shan, Xiaoping Wang

**Affiliations:** 1 Ocean College, Zhejiang University, Zhoushan 316021, China; hycai@zju.edu.cn (H.C.); shannypig@zju.edu.cn (S.S.); 2 Key Laboratory of Ocean Observation-Imaging Testbed of Zhejiang Province, Zhejiang University, Zhoushan 316021, China; 3 The Engineering Research Center of Oceanic Sensing Technology and Equipment, Ministry of Education, Zhoushan 316021, China

**Keywords:** genetic algorithm, high sensitivity, SPR sensor, multilayer thin film

## Abstract

Surface plasmon resonance (SPR) biosensors consisting of alternate layers of silver (Ag) and TiO_2_ thin film have been proposed as a high sensitivity biosensor. The structure not only prevents the Ag film from oxidation, but also enhances the field inside the structure, thereby improving the performance of the sensor. Genetic algorithm (GA) was used to optimize the proposed structure and its maximum angular sensitivity was 384°/RIU (refractive index unit) at the refractive index environment of 1.3425, which is about 3.12 times that of the conventional Ag-based biosensor. A detailed discussion, based on the finite difference time domain (FDTD) method, revealed that an enhanced evanescent field at the top layer–analyte region results in the ultra-sensitivity characteristic. We expect that the proposed structure can be a suitable biosensor for chemical detection, clinical diagnostics, and biological examination.

## 1. Introduction

Surface plasmon resonance (SPR) biosensors have extensive applications in the fields of medical diagnostics, enzyme detection, and food safety analysis due to their unique abilities for label-free, real-time detection [1,2,3,4,5]. These sensors utilize surface plasmon polarization to monitor the change refractive index (RI) of the detected target, and a minor change of RI will result in a significant shift in SPR signal [6]. Generally, silver (Ag) and gold (Au) have been widely used for SPR sensors as plasmonic material. Au is considered as a good material because it is highly resistant to oxidation and corrosion. However, it has low detection accuracy due to a broader resonance curve [7]. In contrast, Ag has a narrower reflectance curve showing higher accuracy, but is less chemically stable because it should oxidize quickly when exposed to the atmosphere [8]. If some protective layers are used to prevent its oxidation, Ag can be effectively used in SPR sensors. According to the widely accepted definition of the angular sensitivity $S_{\theta }=\partial \theta /\partial n$ (where n is the RI of sensing medium and θ is the resonant angle), the angular sensitivity of the conventional SPR sensor composed of a single metal film using the angular interrogation architecture is only 50–150°/RIU [9]. SPR sensors with low sensitivity will hinder direct label-free analysis (especially for small molecules). To achieve high sensitivity, one method is to improve the adsorption efficiency of the biosensor to the biomolecules, and another method is to improve the sensitivity of the biosensor to the RI changes [10].

Generally, biomolecules have poor attachment on the metal surface. In order to improve the attachment of molecules, several surface chemistry methods have been used to attach molecules to the metal surface. For instance, a self-assembled monolayer (SAM) or polymer film is used as a more stable sensing layer covering the metal surface for further immobilization of bioreceptors [11]. Although frequently used, both of them cannot ensure the controlled distribution or orientation of bioreceptors. Therefore, many studies have been reported to obtain uniform orientation, high surface coverage, and make analyte binding more accessible. Nitrilotriacetic acid (NTA) SAMs are widely used for oriented protein immobilization [12]. Based on this method, a high density of oriented protein bioreceptors is covered on the SPR chip, and the limit of detection (LOD) for small molecule drugs is 14 nM [13]. However, this approach is limited to binding to specific proteins.

2D materials such as graphene, transition metal dichalcogenides (TMDCs), and black phosphorous (BP) are also used as a biomolecular recognition element (BRE) on a chip-SPR platform to increase the adsorption of biomolecules [14,15]. Among these materials, graphene has attracted the most attention due to its superior functions including large surface area, charge carrier mobility, and rich π conjugation structure. Singh et al. [16] used a functionalized single graphene layer on a thin gold film to amplify the SPR signal and the LOD for specific antibody anticholera toxin was 4 pg mL^−1^. However, the 2D material had a large extinction coefficient, which may cause unnecessary energy loss, resulting in a wider SPR curve and decreasing the depth of dip.

In addition, numerous efforts have also been devoted to enhance the sensitivity to RI change in a SPR sensor. Researchers have proposed biosensors based on metallic nanoslits [17] and nanoholes [18,19] to improve sensing performances. The local EM field enhancement near nanostructure causes improved sensitivity. In addition, some researchers have investigated biosensors based on hyperbolic metamaterials (HMM) [20,21]. This sensing substrate, with hyperbolic dispersion properties, has been confirmed to significantly improve the performance of the SPR sensors. For instance, Sreekanth et al. [21] fabricated an HMM-based sensor consisting of alternating Au and Al_2_O_3_ layers and achieved a sensitivity of 30,000 nm/RIU and a figure of merit (FOM) of 590. However, the fabrication of these structures involves reactive ion etching or electron beam lithography, which is time-consuming, expensive, and only fabricated in small areas. Moreover, the accurate control of the geometry and optical properties of nanostructures is challenging. Besides the ordered nanoarrays, the disordered system without a delicate structure design also has good prospects for sensors. Garoli et al. [22] exploited nanoporous gold (NPG) as a high-performance sensing platform. Due to the higher surface/volume ratio, these porous materials exhibit extremely high sensitivity. Especially in the near-infrared (NIR), the sensing platform has shown high sensitivity close to 15,000 nm/RIU.

Apart from nanostructures significantly altering the sensing performance, the use of different metal oxides or high RI silicon (Si) over the metal layer can also greatly improve the sensitivity of the biosensor. Bhatia et al. [23] used the high RI of Si material to coat the Ag surface to improve the sensitivity of the SPR sensor. This is because the Si layer improves the intensity of the evanescent field at the Si–analyte interface [24,25]. However, due to the formation of an oxide layer on the surface of Si, the device performance may deteriorate. In contrast, titanium dioxide (TiO_2_) has chemical stability and high RI, which is expected to replace the use of Si in sensing applications. In addition, the use of low RI prism [26] has been proposed to further improve the performance of the biosensor. However, a lower RI prism will reduce the detection range due to the increased resonance angle. Therefore, it is very valuable to propose a SPR biosensor with many advantages such as simple structure, high sensitivity, and low cost.

For SPR sensors with complex structures, performance optimization becomes difficult, which makes the traditional manual optimization methods inefficient. Genetic algorithm (GA) is an efficient global optimization method inspired from the biological evolution process [27]. It is a very powerful tool to deal with the multi-parameter and multi-objective optimization problems. At present, GA is widely used to solve optimization issues of SPR sensors [28].

In this paper, a high sensitivity sensor based on a periodic Ag–TiO_2_ multilayer structure was theoretically investigated. The TiO_2_ layer was used to protect the Ag layer from oxidation. Numerical optimization of sensors was performed using the GA and transfer matrix method (TMM). The result showed that the sensitivity of the sensor reached about 384°/RIU when the RI of the sensing target was 1.3425. Furthermore, the finite-difference time-domain (FDTD) method was used to clearly analyze the physical mechanism that produces the ultra-sensitivity characteristics.

## 2. Structure Design and Optimization

**Figure 1:**
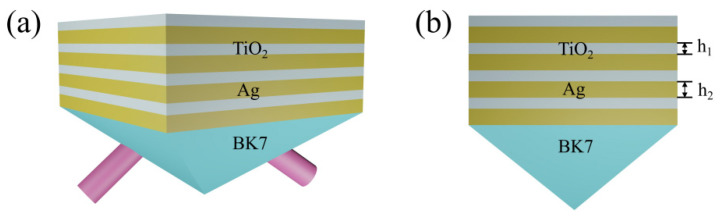
(**a**) 3D schematic diagram of the proposed SPR sensor. (**b**) The corresponding cross-section view.

The proposed SPR biosensor was composed of eight alternating thin films of Ag and TiO_2_, whose configuration is shown in Figure 1a. BK7 glass was selected as the coupling prism, Ag was chosen as the metal layer to excite a surface wave, and TiO_2_ film was used as the dielectric layer to propagate SPP. Figure 1b corresponds to the cross-section view, where $h_{1}$ and $h_{2}$ denote the thicknesses of TiO_2_ and Ag layers, respectively. The operating wavelength (λ) of 633 nm was considered throughout this manuscript. At this wavelength, the BK7 glass had a RI of 1.516 [29]. The RI of TiO_2_ was chosen to 2.41 [30]. The complex RI of Ag is described by the Drude–Lorentz model as follows [31]:

(1) $$n_{m}={\left[1-\frac{\lambda^{2}\lambda_{c}}{\lambda_{p}^{2}(\lambda_{c}+i\lambda )}\right]}^{1/2}$$

where $\lambda_{c}$ and $\lambda_{p}$ represent the collision and the plasma wavelengths, and the numerical values of $\lambda_{c}$ and $\lambda_{p}$ for Ag were 1.7614 × 10^−5^ m and 1.451 × 10^−7^ m, respectively.

Generally speaking, for the performance of SPR sensors, it is required that the sensitivity should be as large as possible, and the full width at half maximum (FWHM) should be as small as possible to achieve reliable resonance sensing. In this paper, a single parameter, combined sensitivity factor (CSF), was used as the performance parameter of the sensor [32,33].

(2) $$\mathrm{CSF}=\frac{\partial \theta_{SPR}}{\partial n_{s}}\times \frac{\left(R_{\max}-R_{\min}\right)}{\mathrm{FWHM}}$$

In the above expression, $\partial n_{s}$ is the change in RI of the sensing medium caused by chemical reaction or biological action, and $\partial \theta_{SPR}$ is the corresponding change in resonance angle. In addition, $R_{\min}$ and $R_{\max}$ represent the normalized reflection values corresponding to the lowest point and highest point of the resonance curve, respectively. From the above expression, an excellent sensor requires a larger CSF value.

**Figure 2:**
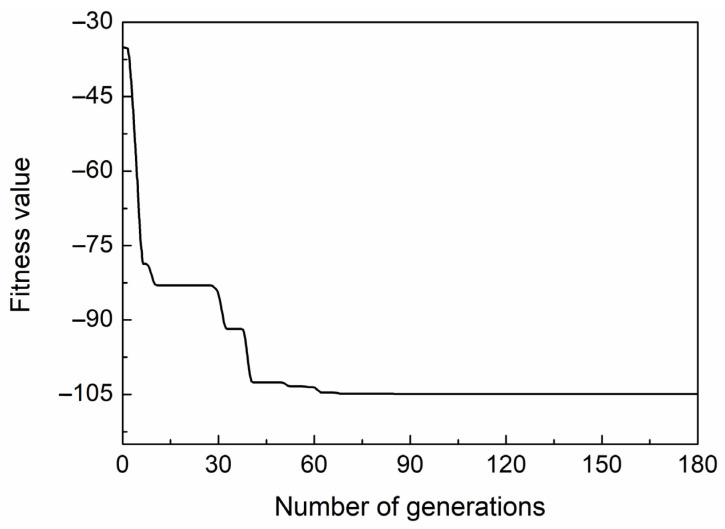
The change in the fitness value versus generation of GA.

In numerical optimization, we used a combination of TMM [34] and GA methods to determine the optimal parameters of the proposed sensor. First, GA randomly generated the original population of structural parameters. Then, TMM was used to calculate the reflectivity curve of the sensor in different refractive index environments. The purpose of the GA optimization was to obtain a minimum value of the fitness function. Therefore, the fitness function was set to -CSF. At the end of each generation, some members with the maximum fitness value are removed by GA. The new offspring are produced through crossover and mutation of the remaining members of population, and are then added to the new population. When the fitness function satisfies the end conditions, the optimization process stops, and the most appropriate population members are found. However, if not, the procedure repeats until the number of iterations is met. A more detailed description of GA can be found in [35]. Figure 2 shows the change in fitness value of each generation in the GA operation. As shown in Figure 2, the fitness function rapidly decreased to a relatively stable value at 90 generations. To ensure the accuracy of these results, the iterations were continued up to 180 generations. Finally, we obtained the value of the fitness function of −104.9 and the best individuals were as follows: $h_{1}$ = 7.7 nm and $h_{2}$ = 11.2 nm.

## 3. Results and Discussion

**Figure 3:**
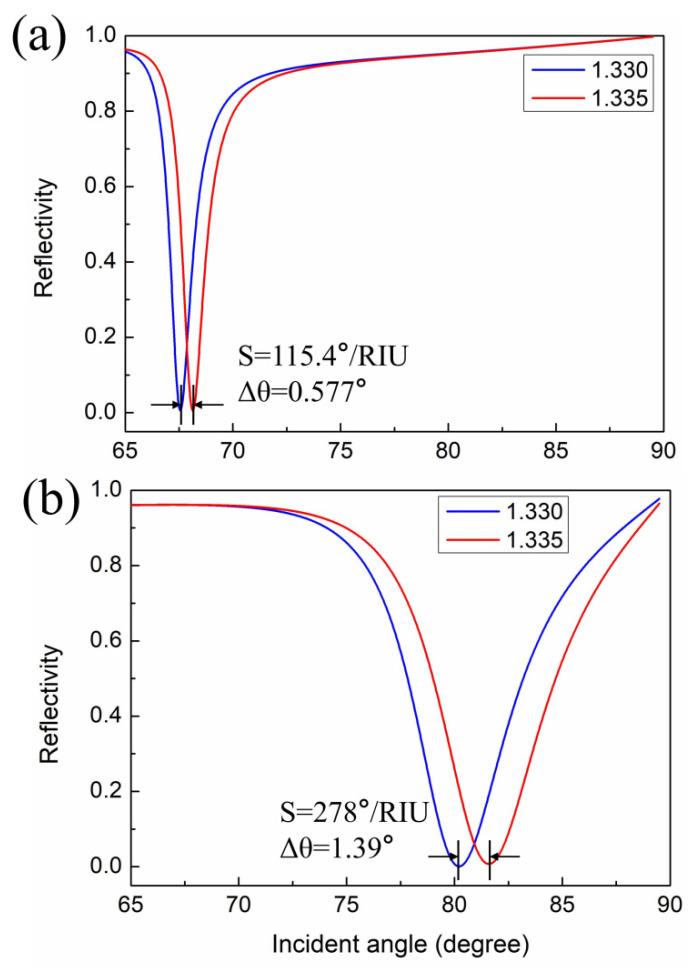
The change in resonance curves versus the angle of incidence for (**a**) conventional SPR sensor; (**b**) the proposed SPR sensor.

In our simulations, the RI for the surrounding detected medium was *n_s_* = 1.330 + Δ*n_s_*, where Δ*n_s_* refers to the change in the RI of the surrounding detected medium. Figure 3 shows the resonance curve for different sensor configurations and the Δ*n_s_* was set to 0.005. The results show that as the RI of analyte increases, the resonance angle shifts to higher values. Figure 3a is the reflectivity curve of a conventional SPR sensor with an Ag thin film thickness of 50 nm. It has been shown that the sensitivity of this sensor was 115.4°/RIU, but was still not high enough to detect minor changes of the sensing targets. Figure 3b is the resonance curve of the sensor based on the optimized geometry. The result shows that the sensitivity was greatly improved, with a sensitivity up to 278°/RIU. The high sensitivity can be attributed to the fact that the periodic multilayer structure increases the light propagation distance within the structure. Thus, most of the incident energy is transferred to the free electrons on the plasmonic metal, so more surface plasmons are generated, resulting in higher sensitivity [36,37]. All in all, in this structure, first, compared with the traditional SPR sensor, the sensitivity was improved several times, and second, the stability of the Ag layer was improved because the top TiO_2_ acted as a protective layer of Ag, which suffers from poor chemical stability.

**Figure 4:**
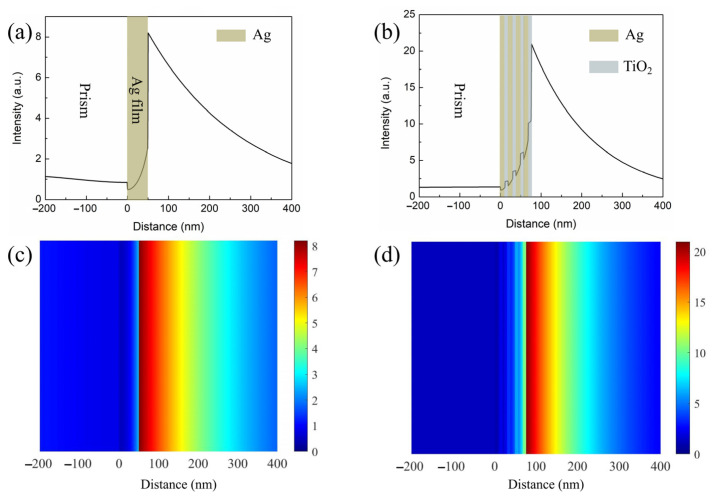
Electric field distributions for (**a**) conventional sensor based on single Ag film, (**b**) the proposed sensor. Two dimensional plots of electric field distribution for (**c**) conventional Ag-based sensor, (**d**) the proposed sensor.

It is well known that the performance of the SPR sensors is largely affected by the SP field distribution on the interface of the sensing medium. Enhancing the evanescent field at the interface of the sensing medium is a direct way to improve the performance of the SPR biosensor [38,39]. The field distribution of the proposed structure was studied by using a finite difference time domain (FDTD) method. Electric field intensity as a function of the distance along the prism to the sensing medium is represented in Figure 4a,b. Moreover, two dimensional plots of the electric field distribution are also illustrated in Figure 4c,d. Obviously, the electric field of the proposed sensor at the interface of the sensing medium is greater than that of a conventional Ag-based biosensor. In the periodic Ag–TiO_2_ multilayer structure, the incident light intensity is first enhanced by the bottom Ag–TiO_2_ layer, and finally gains a great amplification in the top TiO_2_ layer. It is seen that the enhancement of the electric field strength leads to an increased sensitivity.

**Figure 5:**
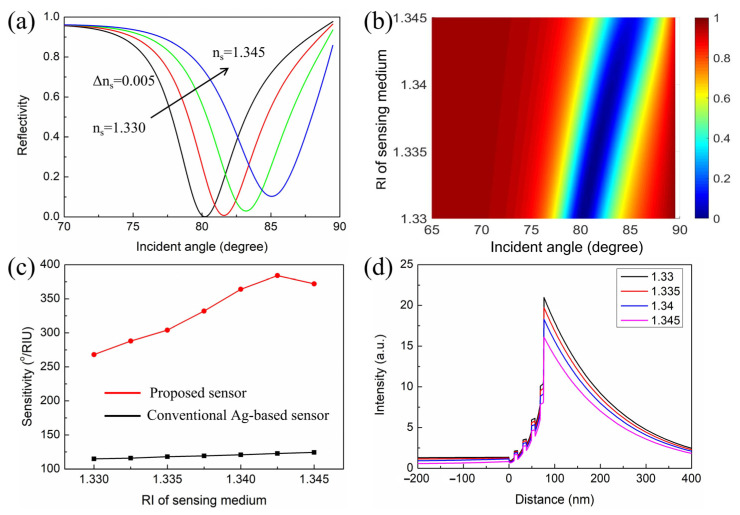
(**a**) The change in resonance curve with respect to the incident angle with *n_s_* = 1.33 to *n_s_* = 1.345. (**b**) Contour map of resonance curve as a function of incident angle and RI of the sensing medium. (**c**) Change in sensitivity with respect to the RI of the sensing medium for different sensors. (**d**) Electric field distributions of the proposed sensor with a change in IR of the sensing medium.

Figure 5a is the SPR spectra for the proposed sensor in RI varying from 1.330 to 1.345, with a step of 0.005. Each curve has a dip at a particular angle known as the resonance angle. With the increases in RI, the SPR curve moved toward the higher angle side. This is due to the increase in the propagation wave vector of the surface plasmon waves (SPWs) [40,41,42]. In addition, the resonance angles were 80.21°, 81.6°, 83.19°, and 85.06° for *n_s_* = 1.33, *n_s_* = 1.335, *n_s_* = 1.34, and *n_s_* = 1.345, respectively. Figure 5b indicates the resonance angle shift corresponded to the RI of the sensing medium varying from 1.33 to 1.345. As shown in Figure 5b, there was a good linear relationship between resonance angle and the RI of the sensing medium. Therefore, the structure still maintained a relatively stable sensing performance in a wide RI span. In addition, we also compared the sensitivity between the proposed SPR sensor and the conventional Ag-based sensor (see Figure 5c). The results showed that the proposed SPR sensor had higher sensitivity than the conventional Ag-based sensor when the RI of the sensing medium varied from 1.33 to 1.345. The maximum sensitivity of the proposed SPR sensor could be obtained at the refractive index environment of 1.3425. It is worth noting that when the RI of the sensing medium exceeded 1.3425, the sensitivity began to decrease. This is because when the RI of the sensing medium was equal to 1.3425, the resonance angle was already up to 83.19°. As the RI continuously increases, the resonance angle will move to 90°, but the detection angle cannot reach 90° [43]. Therefore, the sensitivity will decrease. In addition, corresponding to these resonance angles in Figure 5a, the electric field distributions of the proposed sensor are shown in Figure 5d. The electric field is concentrated at the top TiO_2_–sensing medium, suggesting a strong SP excitation. Furthermore, as the RI of the sensing medium varied from 1.330 to 1.345, the intensity of electric field changed significantly, indicating that the proposed SPR sensor is very sensitive to minor changes in RI.

Considering fabrication feasibility, we further investigated the fabrication tolerance of the proposed SPR sensor in simulations. The fabrication errors were defined as ±10 variations of layer thickness. The sensitivities of the proposed SPR sensor at the refractive index environment of 1.3425 with different fabrication errors were calculated, and the results are shown in Table 1. The fabrication tolerance of the Ag layer was larger than that of the TiO_2_ layer. For this structure, in the actual manufacturing process, the thickness of the TiO_2_ layer above the required thickness causes a sharp decrease in sensitivity. This is because the increase in the thickness of the TiO_2_ layer will cause the resonance angle to move quickly to a high angle. Due to the limitation of the angle range, the sensitivity drops sharply.

**Figure 6:**
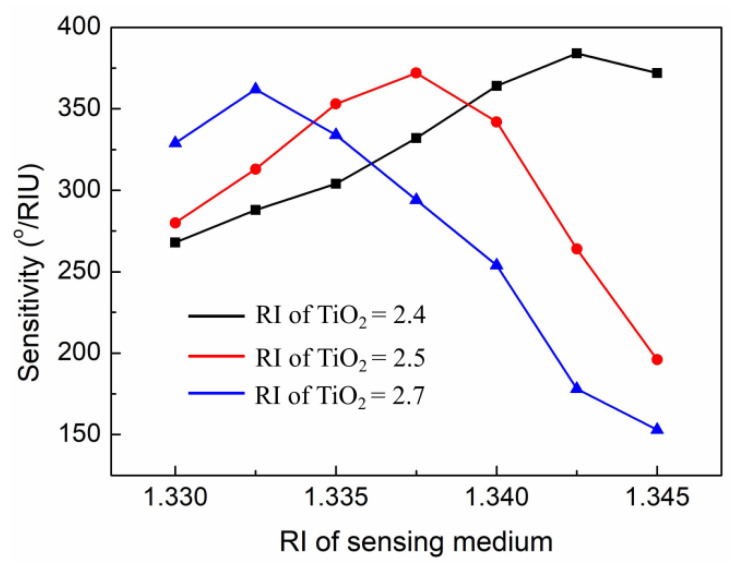
Change in sensitivity with respect to the different RIs of TiO_2_ for the proposed sensor.

In addition, the RI of the material was also affected by the fabrication process. For example, the material of TiO_2_ is known to have two main phases, anatase and rutile, and the corresponding RI are about 2.5 and 2.7, respectively [44]. We calculated the sensitivity of the proposed SPR sensor with different RIs of TiO_2_ and the results are shown in Figure 6. The results show that the RI of TiO_2_ had a great effect on the change in sensitivity. As the RI of TiO_2_ increases, the maximum sensitivity shifts to a low RI of the sensing medium. At the high RI of the sensing medium, the sensitivity drops sharply (especially for anatase and rutile). This is due to increased RI of TiO_2_, leading to higher resonance angle, which limits the sensitivity. In sum, in the actual manufacturing process, the deposition control of TiO_2_ is very important for the proposed SPR sensor.

Finally, we provide a table showing the performance comparisons between the proposed SPR sensor and other existing sensors in the literature. The sensitivity, metal type, and operating wavelength are considered in Table 2. The result shows that the sensitivity obtained here was much higher than all the previously proposed configurations.

## 4. Conclusions

The high-sensitivity SPR sensor based on a periodic Ag–TiO_2_ multilayer structure is theoretically optimized by GA to obtain good sensing performance. Compared to the conventional Ag-based biosensor, the sensitivity of the proposed SPR sensor was as high as 384°/RIU at the refractive index environment of 1.3425. The Ag–TiO_2_ multilayer can effectively couple the light and finally lead to a significant amplification of the electric field on the top TiO_2_ layer. The proposed structure may provide a new approach for the design of ultra-sensitive SPR biosensors.

## Figures and Tables

**Table 1:** The sensitivity of proposed SPR sensor at the refractive index environment of 1.3425 with different fabrication errors.

Layer Thickness	Fabrication Errors	
	−10 Variations	+10 Variations
Ag	372°/RIU	353°/RIU
TiO_2_	323°/RIU	284°/RIU

**Table 2:** The sensitivity, metal type, and operating wavelength for all sensor structures.

Reference	Publication Year	Operating Wavelength	Metal	Sensitivity (Degree/RIU)
[8]	2020	633 nm	Ag	264
[25]	2020	633 nm	Al	148.2
[37]	2019	633 nm	Au	175
[39]	2017	633 nm	Ag	279
[40]	2019	633 nm	Ag	257
[41]	2016	632 nm	Rh and Ag	220
[42]	2019	633 nm	Au	198
[43]	2018	532 nm	Au	224.5
This work	——	633 nm	Ag	384

## Data Availability

The data presented in this study are available on request from the corresponding author upon reasonable request.

## References

[1] Sun Y., Cai H., Wang X., Zhan S. (2020). Layer analysis of axial spatial distribution of surface plasmon resonance sensing. Anal. Chim. Acta.

[2] Daware K., Kasture M., Kalubarme R., Shinde R., Patil K., Suzuki N., Terashima C., Gosavi S., Fujishima A. (2019). Detection of toxic metal ions Pb^2+^ in water using SiO_2_@Au core-shell nanostructures: A simple technique for water quality monitoring. Chem. Phys. Lett..

[3] Zhou J., Qi Q., Wang C., Qian Y., Liu G., Wang Y., Fu L. (2019). Surface plasmon resonance (SPR) biosensors for food allergen detection in food matrices. Biosens. Bioelectron..

[4] Zhou J., Wang Y., Qian Y., Zhang T., Zheng L., Fu L. (2020). Quantification of shellfish major allergen tropomyosin by SPR biosensor with gold patterned Biochips. Food Control.

[5] Chiu N., Yang H. (2020). High-Sensitivity Detection of the Lung Cancer Biomarker CYFRA21-1 in Serum Samples Using a Carboxyl-MoS_2_ Functional Film for SPR-Based Immunosensors. Front. Bioeng. Biotechnol..

[6] Choi S.H., Kim Y.L., Byun K.M. (2011). Graphene-on-silver substrates for sensitive surface plasmon resonance imaging biosensors. Opt. Express.

[7] Maurya J.B., Prajapati Y.K., Singh V., Saini J.P., Tripathi R. (2015). Performance of graphene–MoS_2_ based surface plasmon resonance sensor using Silicon layer. Opt. Quant. Electron..

[8] Kumar R., Pal S., Verma A., Prajapati Y.K., Saini J.P. (2020). Effect of silicon on sensitivity of SPR biosensor using hybrid nanostructure of black phosphorus and MXene. Superlattice. Microst..

[9] Homola J., Koudela I., Yee S.S. (1999). Surface plasmon resonance sensors based on diffraction gratings and prism couplers: Sensitivity comparison. Sens. Actuators. B Chem..

[10] Lin C., Chen S. (2019). Design of highly sensitive guided-wave surface plasmon resonance biosensor with deep dip using genetic algorithm. Opt. Commun..

[11] Altintas Z., Uludag Y., Gurbuz Y., Tothill I. (2012). Development of surface chemistry for surface plasmon resonance based sensors for the detection of proteins and DNA molecules. Anal. Chim. Acta.

[12] Ahijado-Guzmán R., Prasad J., Rosman C., Henkel A., Tome L., Schneider D., Rivas G., Sönnichsen C. (2014). Plasmonic Nanosensors for Simultaneous Quantification of Multiple Protein–Protein Binding Affinities. Nano Lett..

[13] Wang X., Liu Q., Tan X., Liu L., Zhou F. (2019). Covalent affixation of histidine-tagged proteins tethered onto Ni-nitrilotriacetic acid sensors for enhanced surface plasmon resonance detection of small molecule drugs and kinetic studies of antibody/antigen interactions. Analyst.

[14] Duan Q., Liu Y., Chang S., Chen H., Chen J. (2021). Surface Plasmonic Sensors: Sensing Mechanism and Recent Applications. Sensors.

[15] Qu J., Dillen A., Saeys W., Lammertyn J., Spasic D. (2020). Advancements in SPR biosensing technology: An overview of recent trends in smart layers design, multiplexing concepts, continuous monitoring and in vivo sensing. Anal. Chim. Acta.

[16] Singh M., Holzinger M., Tabrizian M., Winters S., Berner N.C., Cosnier S., Duesberg G.S. (2015). Noncovalently Functionalized Monolayer Graphene for Sensitivity Enhancement of Surface Plasmon Resonance Immunosensors. J. Am. Chem. Soc..

[17] Lee K.L., Lee C.W., Wang W.S., Wei P.K. (2007). Sensitive biosensor array using surface plasmon resonance on metallic nanoslits. J. Biomed. Opt..

[18] Stewart M.E., Anderton C.R., Thompson L.B., Maria J., Gray S.K., Rogers J.A., Nuzzo R.G. (2008). Nanostructured Plasmonic Sensors. Chem. Rev..

[19] Sherry L.J., Jin R., Mirkin C.A., Schatz G.C., Van Duyne R.P. (2006). Localized Surface Plasmon Resonance Spectroscopy of Single Silver Triangular Nanoprisms. Nano Lett..

[20] Poddubny A., Iorsh I., Belov P., Kivshar Y. (2013). Hyperbolic metamaterials. Nat. Photonics.

[21] Sreekanth K.V., Alapan Y., ElKabbash M., Ilker E., Hinczewski M., Gurkan U.A., De Luca A., Strangi G. (2016). Extreme sensitivity biosensing platform based on hyperbolic metamaterials. Nat. Mater..

[22] Garoli D., Calandrini E., Giovannini G., Hubarevich A., Caligiuri V., De Angelis F. (2019). Nanoporous gold metamaterials for high sensitivity plasmonic sensing. Nanoscale Horiz..

[23] Bhatia P., Gupta B.D. (2011). Surface-plasmon-resonance-based fiber-optic refractive index sensor: Sensitivity enhancement. Appl. Opt..

[24] Lahav A., Auslender M., Abdulhalim I. (2008). Sensitivity enhancement of guided-wave surface-plasmon resonance sensors. Opt. Lett..

[25] Su M., Chen X., Tang L., Yang B., Zou H., Liu J., Li Y., Chen S., Fan D. (2020). Black phosphorus (BP)–graphene guided-wave surface plasmon resonance (GWSPR) biosensor. Nanophotonics.

[26] Wu L., Jia Y., Jiang L., Guo J., Dai X., Xiang Y., Fan D. (2017). Sensitivity Improved SPR Biosensor Based on the MoS_2_/Graphene–Aluminum Hybrid Structure. J. Lightwave Technol..

[27] Kundu P.K., Elkamel A., Vargas F.M., Farooq M.U. (2018). Genetic algorithm for multi-parameter estimation in sorption and phase equilibria problems. Chem. Eng. Commun..

[28] Lin C., Chen S. (2019). Design of high-performance Au-Ag-dielectric-graphene based surface plasmon resonance biosensors using genetic algorithm. J. Appl. Phys..

[29] Zeng S., Hu S., Xia J., Anderson T., Dinh X., Meng X., Coquet P., Yong K. (2015). Graphene–MoS_2_ hybrid nanostructures enhanced surface plasmon resonance biosensors. Sens. Actuators B Chem..

[30] Cai H., Sun Y., Wang X., Zhan S. (2020). Design of an ultra-broadband near-perfect bilayer grating metamaterial absorber based on genetic algorithm. Opt. Express.

[31] Gupta B.D., Sharma A.K. (2005). Sensitivity evaluation of a multi-layered surface plasmon resonance-based fiber optic sensor: A theoretical study. Sens. Actuators B Chem..

[32] Abbas A., Linman M.J., Cheng Q. (2011). Sensitivity comparison of surface plasmon resonance and plasmon-waveguide resonance biosensors. Sens. Actuators B Chem..

[33] Zhou Y., Zhang P., He Y., Xu Z., Liu L., Ji Y., Ma H. (2014). Plasmon waveguide resonance sensor using an Au-MgF_2_ structure. Appl. Opt..

[34] Haddouche I., Cherbi L. (2017). Comparison of finite element and transfer matrix methods for numerical investigation of surface plasmon waveguides. Opt. Commun..

[35] Nguyen-Huu N., Lo Y.L., Chen Y.B., Yang T.Y. (2011). Realization of integrated polarizer and color filters based on subwavelength metallic gratings using a hybrid numerical scheme. Appl. Opt..

[36] Das C.M., Ouyang Q., Kang L., Guo Y., Dinh X., Coquet P., Yong K. (2020). Augmenting sensitivity of surface plasmon resonance (SPR) sensors with the aid of anti-reflective coatings (ARCs). Photonics Nanostructures Fundam. Appl..

[37] Zhao Y., Gan S., Zhang G., Dai X. (2019). High sensitivity refractive index sensor based on surface plasmon resonance with topological insulator. Results Phys..

[38] Feng Y., Liu Y., Teng J. (2018). Design of an ultrasensitive SPR biosensor based on a graphene-MoS_2_ hybrid structure with a MgF_2_ prism. Appl. Opt..

[39] Wu L., Guo J., Wang Q., Lu S., Dai X., Xiang Y., Fan D. (2017). Sensitivity enhancement by using few-layer black phosphorus-graphene/TMDCs heterostructure in surface plasmon resonance biochemical sensor. Sens. Actuators B Chem..

[40] Sun P., Wang M., Liu L., Jiao L., Du W., Xia F., Liu M., Kong W., Dong L., Yun M. (2019). Sensitivity enhancement of surface plasmon resonance biosensor based on graphene and barium titanate layers. Appl. Surf. Sci..

[41] Mishra A.K., Mishra S.K. (2016). Gas sensing in Kretschmann configuration utilizing bi-metallic layer of Rhodium-Silver in visible region. Sens. Actuators B Chem..

[42] Xu Y., Ang Y., Wu L., Ang L. (2019). High Sensitivity Surface Plasmon Resonance Sensor Based on Two-Dimensional MXene and Transition Metal Dichalcogenide: A Theoretical Study. Nanomaterials.

[43] Wu L., You Q., Shan Y., Gan S., Zhao Y., Dai X., Xiang Y. (2018). Few-layer Ti_3_C_2_T_x_ MXene: A promising surface plasmon resonance biosensing material to enhance the sensitivity. Sens. Actuators B Chem..

[44] Wicaksana D., Kobayashi A., Kinbara A. (1992). Process effects on structural properties of TiO_2_ thin films by reactive sputtering. J. Vac. Sci. Technol. A.

